# Use of bone landmarks for assessing the safety of acupuncture on the posterior midline of the neck region

**DOI:** 10.1186/s12906-024-04466-6

**Published:** 2024-04-22

**Authors:** Zhiliang Tang, Jiao Liu, Jin Li, Chunming Ma

**Affiliations:** Department of Human Anatomy, School of Basic Medicine Sciences, Weifang Medical University, 7166 Baotongxi Street, Weicheng District, Weifang, 261053 Shandong China

**Keywords:** Cervical vertebrae, Spinous process, Acupuncture, Mastoid process, Anatomy

## Abstract

**Objective:**

Many acupuncture acupoints are located on the posterior midline of the neck region. The needling depth for acupuncture is important for practitioners, and an unsafe needling depth increases the possibility of damage to the spinal cord and brainstem. Can the safety of acupuncture be assessed by examining bone structures? We focused on this aim to carry out this study.

**Methods:**

The shortest distance from the posterior border of the foramen magnum to the line joining both upper ends of the posterior border of the mastoid process was measured on 29 skulls. Distances from the posterior border of the vertebral foramen to the tip of the spinous process and posterior tubercle of the atlas were measured and evaluated from 197 dry cervical vertebrae and 31 lateral cervical radiographs of patient subjects. The anatomic relationships of the vertebral canal with the external occipital protuberance, tip of the spinous process of the axis, tip of the posterior tubercle of the atlas, and upper end of the posterior border of the mastoid process were observed and evaluated via lateral cervical radiography.

**Results:**

The shortest distance from the foramen magnum to the line between the mastoid processes was 4.65±1.75 mm, and the distance from the superior border of the vertebral foramen of the atlas to the posterior tubercle was less than the distance from the inferior border. The distance from the superior border of the vertebral canal to the tip of the spinous process in C2-C7 was greater than the distance from the inferior border. The mean lengths of the superior border of the C2 spinous process and the inferior border of the C7 spinous process were greater than 21 mm and 31 mm, respectively. The line from the upper end of the posterior border of the mastoid process to the tip of the C2 spinous process or 10 mm deep to the tip of the C2 spinous process was posterior to the vertebral canal.

**Conclusions:**

On the posterior midline of the neck region between the tip of spinous process of axis and external occipital protuberance, if the needle reaches the depth of the line between the upper end of posterior border of mastoid process and the tip of the spinous process of the axis, approximately 10 mm along the spinous process of the axis, the needle is in the safe region. The mean length of the C2-C7 spinous process is suitable to accommodate the needling depth of the adjacent acupoint. Bone structures can be used to effectively assess the safety of acupuncture on the posterior midline of the neck region.

## Introduction

Acupuncture is a traditional Chinese medicine technique in which a needle is inserted into the body at a specific point (named the acupoint). Currently, it is popular in many countries [1] and in public health care [2–4]. Acupuncture at the posterior midline of the neck region can treat several disorders of the nervous system [5, 6], digestive system [7], and respiratory system [8, 9], as well as diseases in this region [10]. There are three acupoints in the governor vessel, namely, Fengfu (GV16), Yamen (GV15), and Dazhui (GV14), on the posterior midline between the occipital bone and atlas, atlas and axis, and the seventh cervical vertebral spinous process and first thoracic vertebral spinous process. These three acupoints yield good treatment results [5, 11, 12]. However, in this region, acupuncture is not performed deep beyond the posterior border of the vertebral canal to reach the epidural space to avoid damaging the spinal cord and brainstem.

The safety of acupuncture is the first consideration for acupuncturists [13]. The quantitative anatomy of the spine is very important for acupuncturists. Familiarity with the quantitative data of the normal spine anatomy is a prerequisite for ensuring the safety of acupuncture. Imaging can provide this information and is readily accessible in most hospitals. However, it is not possible for each patient to undergo imaging before or during acupuncture treatment, especially outside the hospital. For novice acupuncturists who do not clearly understand the importance of depth distance determination or who have not identified important landmarks to determine the depth distance, confident needle insertion is unlikely because of the anxiety of damaging the spinal cord or brainstem.

In this study, we aimed to evaluate the safety of acupuncture by identifying the bone structures on the posterior midline of the neck region. First, we investigated and analysed the length of the cervical vertebra from the posterior border of the vertebral foramen to the posterior end of the vertebral arch from dry bone and lateral cervical radiographs of Chinese patients to evaluate whether the length could meet the required depth for acupuncture. Second, we evaluated the relationship between the distance of the upper end of the posterior border of the mastoid process and foramen magnum occipitalis from the dry skull to determine whether the upper end of the posterior border of the mastoid process can be a reliable landmark for ensuring the safety of acupuncture.

## Materials and methods

The ethical review committee of our institution approved this study (2022YX035).

**Fig. 1:**
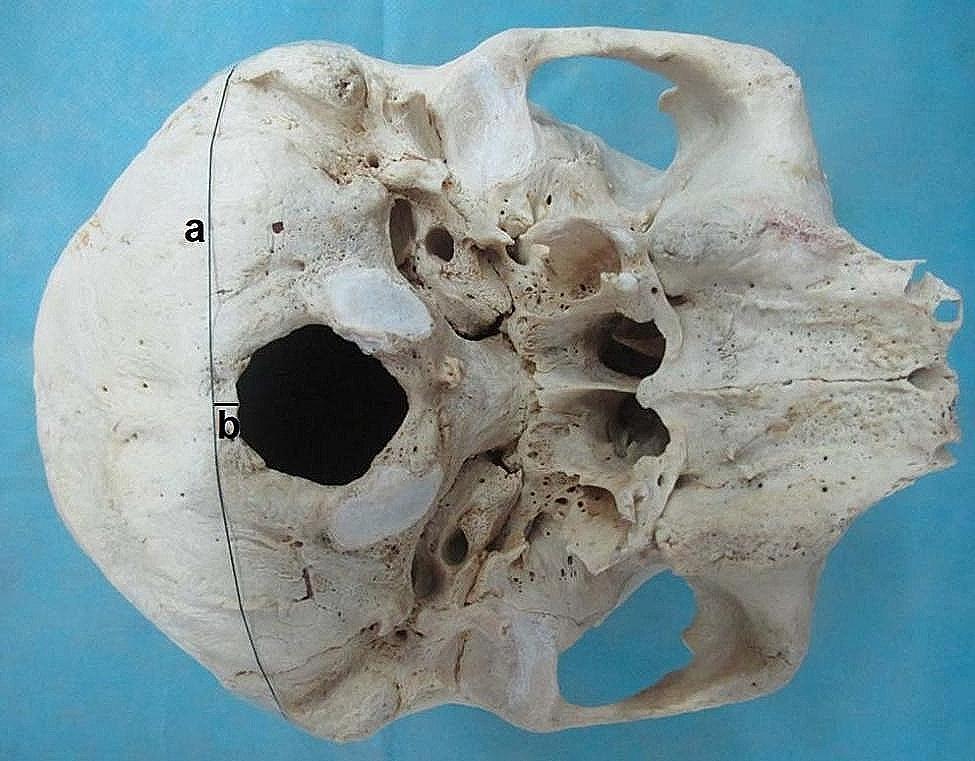
Skull measurement. Surgical suture a was used to connect the bilateral base of the posterior border of the mastoid process. Line b indicates the shortest distance from the posterior border of the foramen magnum to surgical suture a

**Fig. 2:**
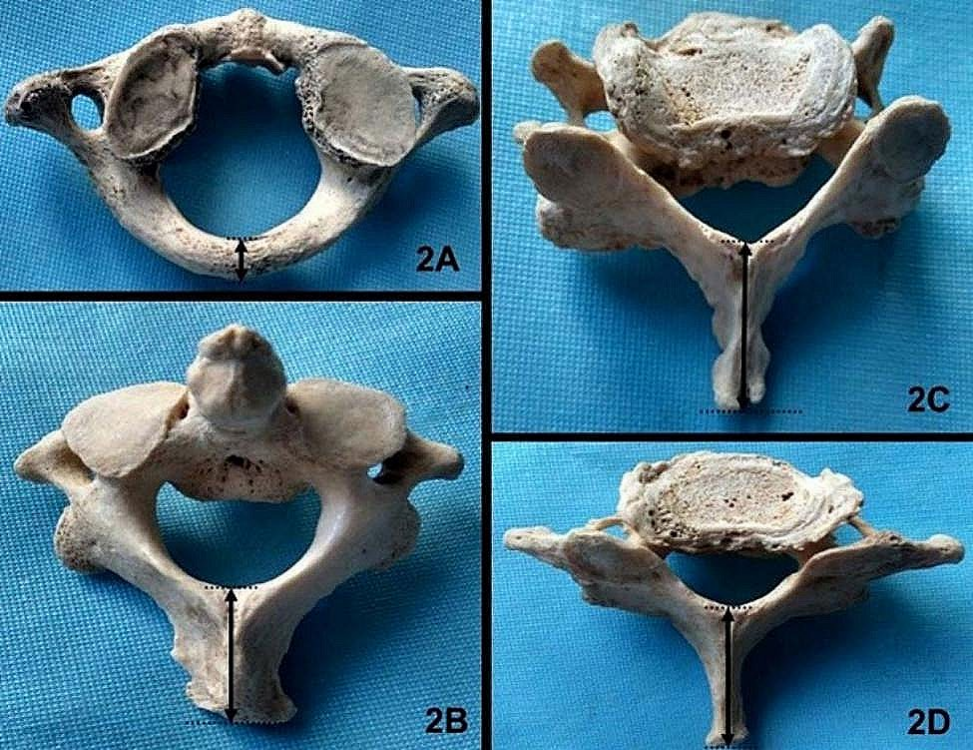
Measurement of cervical vertebrae **2A** shows the distance from the tip of posterior tubercle of atlas to posterior wall of vertebral foramen; **2B** shows the distance from the tip of spinous process of C2 to the anterior wall of the lamina of vertebral arch, the tip of spinous process is asymmetrical; **2C** shows the distance from the tip of spinous process of C6 to the anterior wall of the lamina of vertebral arch, the spinous process deviates from midline; **2D** shows the distance from the tip of spinous process of C7 to the anterior wall of the lamina of vertebral arch, the tip of spinous process is asymmetrical

1 Dry skull and cervical vertebrae from 29 adult cadavers were selected randomly from the anatomy laboratory. The sex of the bone specimens was unknown, and there were no signs of surgery, trauma or other diseases that could alter the shape of the studied bone. One C1 vertebral bone, two C4 vertebral bones, two C6 vertebral bones, and one C7 vertebral bone were excluded from this study due to accidental damage to these bones at the time of measurement. A total of 197 cervical vertebrae (28 C1, 29 C2, 29 C3, 27 C4, 29 C5, 27 C6, 28 C7) were included in this study. Surgical suture was used to connect the bilateral upper end of the posterior border of the mastoid processes, and the shortest distance from the posterior border of the foramen magnum to the surgical suture was measured (Fig. 1). If the posterior border of the foramen magnum was anterior to the surgical suture, the distance was recorded as a positive number; otherwise, it was recorded as a negative number. The distance from the tip of the posterior tubercle of the atlas to the posterior wall of the vertebral foramen and the straight-line distance from the tip of the spinous process of C2-C7 to the anterior wall of the lamina of the vertebral arch on the midline along the superior and inferior border of the spinous process were measured and recorded (Fig. 2). A digital Vernier calliper with an accuracy to 0.01 mm was used for the measurements. Two authors performed the measurements three times, and the average data were recorded.

**Fig. 3:**
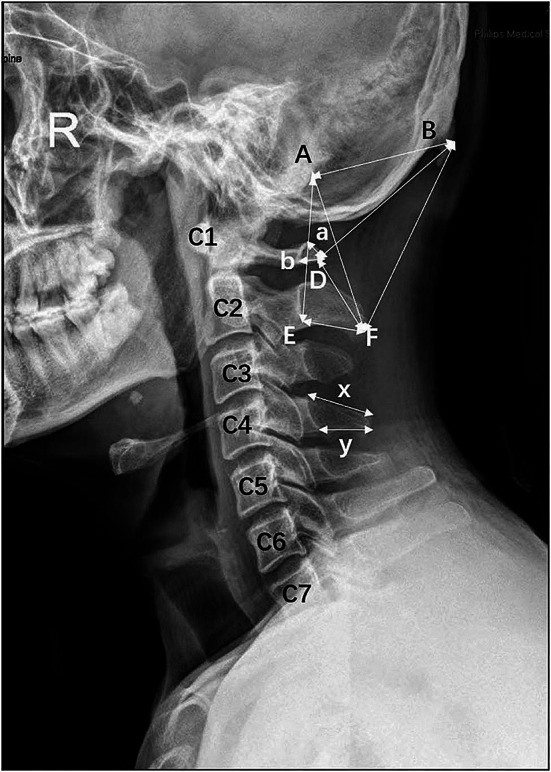
Lateral cervical radiograph. C1- C7 show the line from posterior border of spinal aperture to posterior tubercle of atlas or tip of spinal process; **A**: the base of the posterior border of the mastoid process; **B**: The external occipital protuberance; **D**: the tip of the posterior tubercle of the atlas; **E**: the anterior end of the inferior border of the spinous process of the axis; **F**: the tip of the spinous process of the axis; a: the upper border length from the tip of the posterior tubercle of the atlas to the posterior wall of the vertebral foramen; b: the lower border length from the tip of the posterior tubercle of the atlas to the posterior wall of the vertebral foramen; x: the length of the superior border of the spinous process; y: the length of the inferior border of the spinous process

2 Lateral cervical radiographs (Philips Medical Systems DMC GmbH, Hamburg, Germany) from 31 patients (12 male, age range 31–78 years, average age 40.42 years; 19 female, age range 31–63 years, average age 45.32 years) available from our teaching hospital were used in this study. Written informed consent was obtained from each patient. Lateral cervical radiographs without vertebral rotation and diseases that could alter the shape of the skull and cervical vertebrae were included in this study. On lateral cervical radiographs, two procedures were conducted to collect the data. First, the following distances were measured with the Philips Medical System Digital Diagnost and recorded (Fig. 3). The distance from the tip of the posterior tubercle of the atlas to the posterior wall of the vertebral foramen, and the distance from the tip of the spinous process of C2-C7 to the anterior wall of the lamina of the vertebral arch on the midline along the superior and inferior borders of the spinous process were measured. Second, the upper end of the posterior border of the mastoid process was set as A, the external occipital protuberance was set as B, the tip of the posterior tubercle of the atlas was set as D, the anterior end of the inferior border of the spinous process of the axis was set as E, and the lower end of the tip of the spinous process of the axis was set as F. Lines from B to A, B to D, B to F, A to F, F to D, and F to E (Fig. 3) were drawn. Moreover, the anatomic relationships of the vertebral canal with the external occipital protuberance, the tip of the spinous process of the axis, the tip of the posterior tubercle of the atlas, and the base of the posterior border of the mastoid process were observed.

The measured data were entered into a Microsoft Excel file, are presented as the mean ± standard deviation (mean ± SD) and were analysed by IBM SPSS statistics 27 (IBM Corp., Armonk, NY, USA). A paired samples *t* test was used to compare the average data of each cervical vertebra from dry bony specimens and lateral cervical radiographs and to compare the female and male average data of each cervical vertebra from lateral cervical radiographs. A *p* value of < 0.05 was considered to indicate a statistically significant difference. Moreover, a trend graph of the average values from dry bone and lateral cervical radiographs of the cervical vertebrae was generated, and the female and male average values of the cervical vertebrae from lateral cervical radiographs were graphed.

## Results

The average distance from the posterior border of the foramen magnum to the surgical suture was 4.65±1.75 mm, the minimum distance was 0.80 mm, and the maximum distance was 6.90 mm. None of the measurements were negative.

**Fig. 4:**
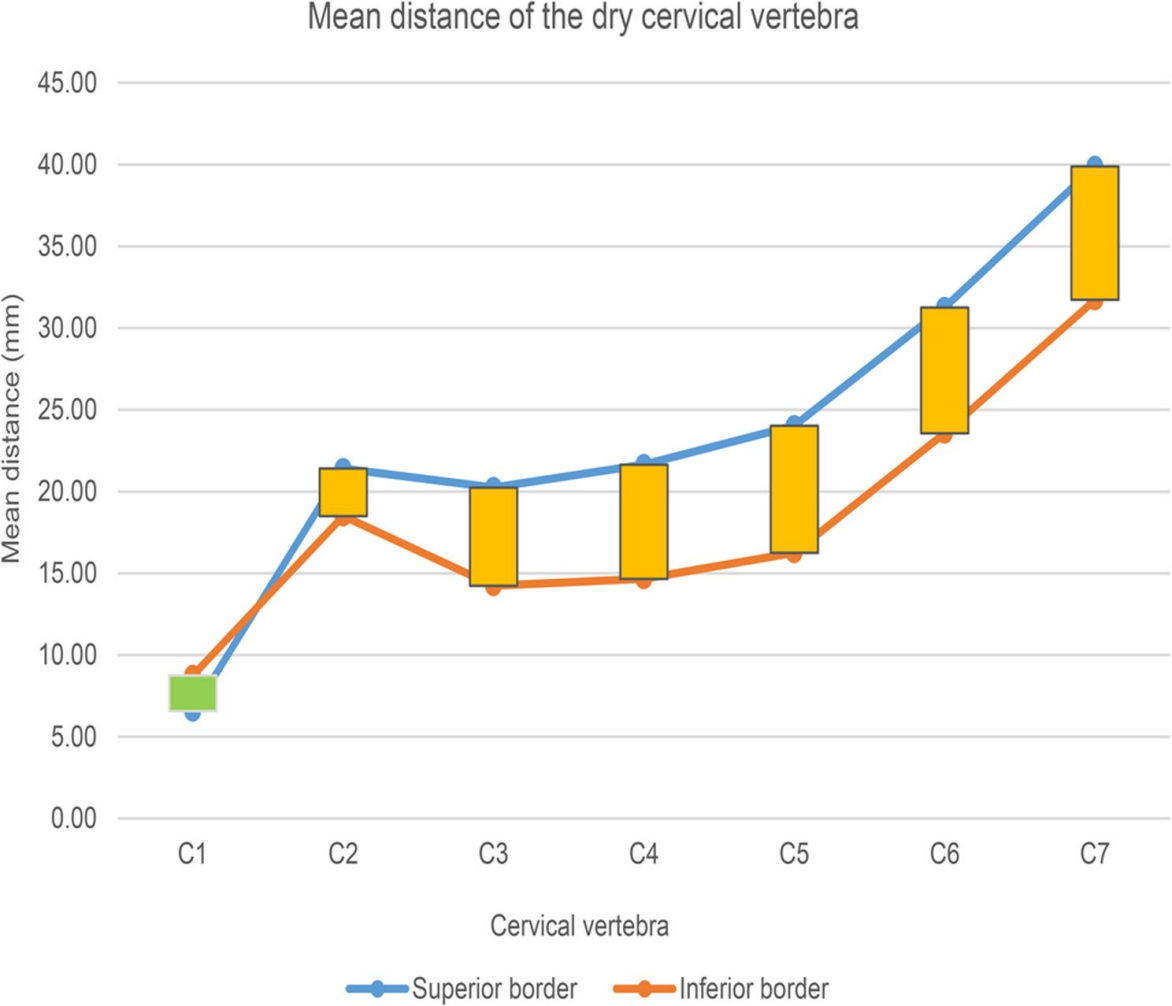
Mean distance of the dry cervical vertebra. The distance from the tip of the posterior tubercle of the atlas to the vertebral canal was short, and the distance from the inferior border was greater than the distance from the superior border. The length of the spinous process increases from C2 to C7, and the length of the superior border of the spinous process is greater than the length of the inferior border in each spinous process. The longest length is observed in C7, and the shortest length is observed in C3. A slight increase is observed from C3 to C5, and a sharp increase is observed from C3 to C7

**Fig. 5:**
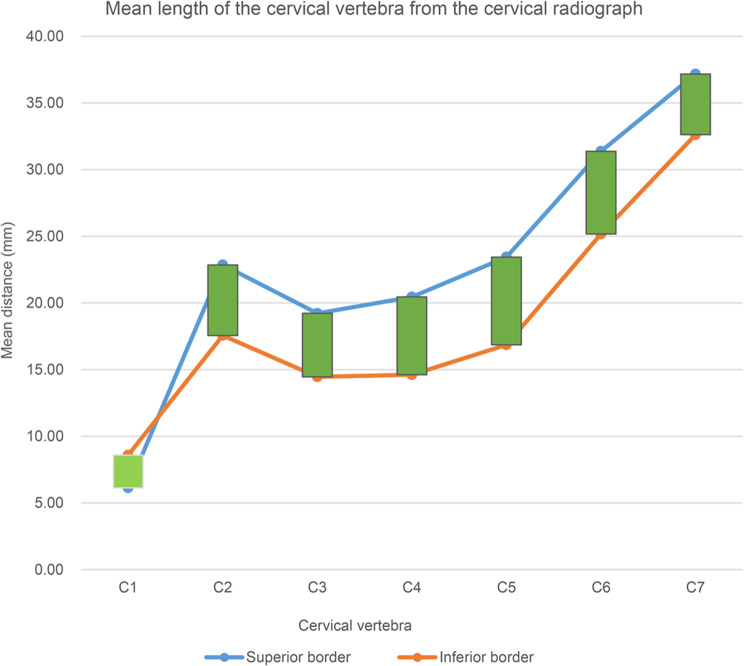
Mean length of the cervical vertebra from the cervical radiograph. The distance from the tip of the posterior tubercle of the atlas to the vertebral canal was short, and the distance from the inferior border was greater than the distance from the superior border. The length of the spinous process increases from C2 to C7, and the length of the superior border of the spinous process is greater than the length of the inferior border in each spinous process. The longest length is observed in C7, and the shortest length is observed in C3. A slight increase is observed from C3 to C5, and a sharp increase is observed from C3 to C7

The average cervical vertebra data from the dry cervical vertebrae and cervical radiographs are shown in Table 1. The trend graph of the average value from the dry bone of the cervical vertebrae is shown in Fig. 4, and that from the lateral cervical radiograph is shown in Fig. 5. The trend in the dry bone density was similar to that in the lateral cervical radiograph. The distance from the tip of the posterior tubercle of the atlas to the vertebral foramen was small, and the distance from the inferior border was greater than the distance from the superior border. The length of the superior border and inferior border of the spinous process from C2 was greater than that from C3, and the length of the spinous process increased from C3 to C7. A slight increase was observed from C3 to C5, and a sharp increase was observed from C5 to C7. The length of the superior border of the spinous process was greater than the length of the inferior border on each spinous process. The maximum length of the spinous process was observed in C7, and the minimum length was observed in C3.

**Table 1:** Measured data of cervical vertebra (mean ± SD, min-max, mm)

		C1	C2	C3	C4	C5	C6	C7
Dry bone	Superior border	6.56 ± 2.43 2.56–11.84	21.40 ± 4.96 15.82–32.36	20.24 ± 4.06 13.34–26.54	21.64 ± 4.53 12.50-30.91	24.02 ± 6.57 14.30-40.49	31.26 ± 6.80 18.89–49.46	39.89 ± 7.55 24.41–62.01
	Inferior border	8.75 ± 3.55 2.32–15.94	18.50 ± 3.37 12.98–28.22	14.24 ± 2.57 9.23–18.45	14.66 ± 3.24 5.54–21.24	16.25 ± 5.24 6.43–28.04	23.57 ± 6.85 11.30-40.86	31.73 ± 7.63 17.34–52.15
Radiograph	Superior border	6.13 ± 1.94 2.61–10.98	22.85 ± 3.57 17.58–31.68	19.22 ± 3.51 11.70-26.99	20.45 ± 3.96 12.25–28.90	23.44 ± 3.50 15.97–31.03	31.37 ± 5.13 20.94–43.57	37.16 ± 4.64 30.25–46.34
	Inferior border	8.58 ± 2.40 3.90-13.51	17.56 ± 3.43 11.87–25.39	14.45 ± 2.57 11.16–20.15	14.61 ± 3.24 9.28–24.72	16.86 ± 3.96 8.96–24.63	25.17 ± 4.73 17.41–34.23	32.62 ± 4.99 23.88–44.13

From the lateral cervical radiograph, point D between the occipital bone and the spinal process of the axis was deep to the BF line. One point was posterior to the foramen magnum and the spinal cord. The mastoid process acted as a cover for the superior part of the vertebral canal. The BF line and AF line were posterior to the vertebral canal and AE line, respectively, and the needle tip that punctured into this space between these two lines cannot injure the spinal cord. Thus, the needle is in a safe zone if the needle tip lies posterior to the line between the upper end of the posterior border of the mastoid process and the tip of the spinous process of the axis. If the needle is inserted to the depth of the AF line and superficial to the AE line (the length of inferior border of spinous process of axis determines the distance from F point to E point and is over 10 mm), posterior to the vertebral canal and spinal cord, it is therefore in a safe region.

**Fig. 6:**
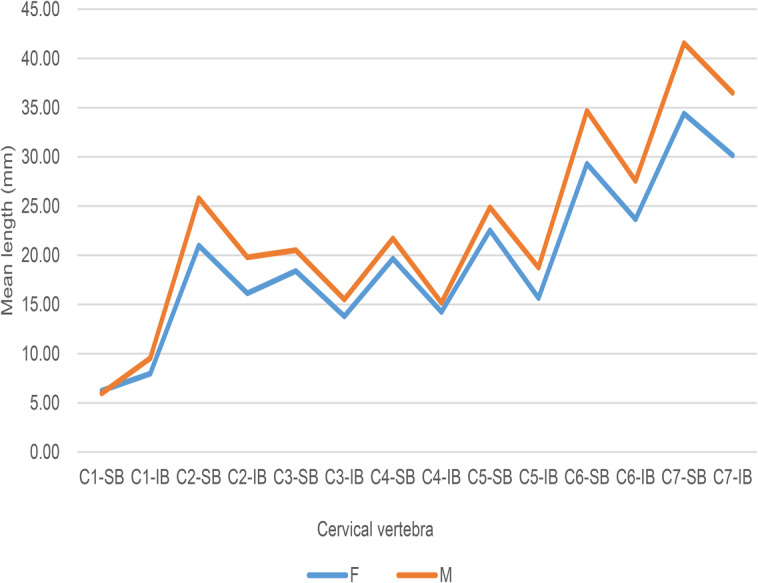
Comparison of the mean measured data from lateral cervical radiographs. The superior border of C1 in a female is greater than that in a male, the other values are lower in females than those in males, and the distance from the inferior border of C4 in females is close to that in males. SB: superior border; IB: inferior border; F: female; M: male

A paired samples *t* test was performed to compare the average measured data from dry bone specimens and lateral cervical radiographs. A *p* value of 0.608 was obtained. There was no statistically significant difference between the dry bone specimen and lateral cervical radiograph data. The lateral cervical radiograph data revealed significant sex differences, with the differences in the average values among males being greater than the differences in the average values among females (*p* < 0.001). Figure 6 reveals that the trend graph for the males is similar to that for the females, however, the superior border of C1 in a female is greater than that in a male, the other values are lower in females than those in males, and the distance from the inferior border of C4 in females is close to that in males.

## Discussion

The tip of the spinous process of the axis can be easily palpated on the body surface [14, 15], and the upper end of the posterior border of the mastoid process is a distinct bony landmark. According to the results of our study, if the needle tip reaches the line between the upper end of the posterior border of the mastoid process and the tip of the spinous process of the axis, the needle is in a safe region. If the needle tip reaches the line between the lower end of the tip of the spinous process of the axis, approximately 10 mm, and the upper end of the posterior border of the mastoid process, the needle tip is posterior to the vertebral canal and in the safe region. Similarly, if the needle tip is approximately 15 mm deep between the upper end of the tip of the spinous process of the axis (lower than the minimum of the measured superior border of the spinous process of C2) and the upper end of the posterior border of the mastoid process, then it is in a safe region.

The needling depth on the posterior midline of the neck region was 0.5 to one cun (measurement unit for acupuncture) according to the Chinese traditional medicine textbook. From a study by Godson DR and Wardle JL [16], thumb cun was 1.99 ± 0.01 cm, middle finger cun, 2.03 ± 0.01 cm, four-finger cun, 2.06 ± 0.01 cm for Chinese individuals. In our study, the mean depth of the inferior border of the C7 spinous process was greater than the needling depth of Dazhui (GV14), and the mean value of the superior border of the C2 spinous process was greater than the needling depth of Yamen (GV15) and Fengfu (GV16). The mean length of the spinous process met the needling depth of the adjacent acupoint along the posterior midline of the neck region. However, the measured minimum depth of the spinous process is less than the needling depth; practitioners should pay attention to this difference in depth to avoid damaging the spinal cord and brainstem.

Many researchers have measured the depth from the skin surface to the posterior midline of the neck region in living people [11–13, 17–19]. In the clinical setting, procedures performed on the posterior midline of the neck region also involved cervical interlaminar midline epidural steroid injection. Many anaesthetists and radiologists are concerned about the distance from the skin to the epidural space after surgical interlaminar epidural steroid injection for herniated disc disease and spinal stenosis [20–22]. The depth of acupuncture and cervical interlaminar midline epidural steroid injection used in published studies involved the thickness of soft tissue from the skin to the underlying layers. The thickness of the soft tissue from the skin to the ligamentum nuchae can be determined by imaging. Another method for obtaining this information is the pinch test. The pinch test is an easy, quick, reliable, and practical manipulation method that is performed with a calliper to obtain the thickness of the pinched structure [23, 24]. However, the mutual relationship between the thickness of the pinched structure on the posterior neck region and the thickness of the soft tissue posterior to the cervical vertebrae is unclear and should be established in future studies. This approach provides feasibility for obtaining the thickness.

The length of the C2-C7 spinous process plus the thickness of the pinched structures posterior to the tip of the spinous process of the cervical vertebrae can determine the needling depth for acupuncture and the cervical interlaminar midline epidural steroid injection and evaluate the safety of these procedures. By understanding the distance in this region, the practitioner can preliminarily judge whether the needle tip lies posterior to the ligamentum flava and whether subsequent manipulation increases the possibility of damaging the spinal cord or brainstem.

Interestingly, the length of the spinous process has different definitions. Leng LN et al. [25] determined the length from the posterior aspect of the lamina of the vertebral arch along the spinous process. Tan SH et al. [26, 27] and Panjabi MM et al. [28, 29] reported that the length of the spinous process ranged from the tip of the spinous process to the centre of the upper surface of the vertebral body. In our study, we obtained the length from the tip of the spinous process to the vertebral foramen along the upper border and lower border of the spinous process; furthermore, we quantitatively evaluated the characteristics of the Chinese anatomy. The measurements of C3-C7 recorded by Tan SH et al. [26] are greater than those in our study, and the variability of these values are similar to our results. The measurements of C2-C7 recorded in a study by Panjabi MM et al. [28] are greater than those recorded in our study; however, the variability of the values is somewhat similar to that of lateral cervical radiography, and the measurement of C5 is the lowest among C2-C7.

## Limitations

The small sample size was one of our limitations. Additionally, this was not a multiple-centre experimental study. Similar studies should be conducted to evaluate the quantitative data and anatomic relationships in other regions of the world and to determine whether bone structure data can be used to assess the safety of acupuncture for needling depth.

## Conclusions

On the posterior midline of the neck region between the tip of the C2 spinous process and the external occipital protuberance, the needle tip reaches the line between the upper end of the posterior border of the mastoid process and the tip of the C2 spinous process, and the needle is in a safe region. If the needle tip reaches the line between the deep to lower end of the tip of the C2 spinous process approximately 10 mm and the upper end of the posterior border of the mastoid process, the needle tip is posterior to the vertebral canal and in the safe region. Similarly, between the position approximately 15 mm deep to the upper end of the tip of the C2 spinous process and the upper end of the posterior border of the mastoid process, the needle tip is also in a safe region. The mean length of the C2-C7 spinous process met the needling depth of the adjacent acupoint along the posterior midline of the neck region. The mean length of the C2-C7 spinous process plus the thickness of the soft tissue posterior to the cervical vertebrae can accommodate the needling depth for acupuncture, and the thickness of the structures posterior to the cervical vertebrae can be obtained by imaging and pinching tests. By understanding the distance, the practitioner can preliminarily judge whether the needle tip lies posterior to the ligamentum flava and whether subsequent manipulation increases the possibility of damaging the spinal cord or brainstem.

## Data Availability

The datasets used and analysed during the current study are available from the corresponding author on reasonable request.
